# Exploration of Site-Specific Drug Targeting—A Review on EPR-, Stimuli-, Chemical-, and Receptor-Based Approaches as Potential Drug Targeting Methods in Cancer Treatment

**DOI:** 10.1155/2022/9396760

**Published:** 2022-09-29

**Authors:** Md. Shamiul Islam Rasel, Farhana Afrin Mohona, Wahida Akter, Shaila Kabir, Abu Asad Chowdhury, Jakir Ahmed Chowdhury, Md. Abul Hassan, Abdullah Al Mamun, Dipayon Krisna Ghose, Zubair Ahmad, Farhat S. Khan, Md. Fazlul Bari, Md. Sohanur Rahman, Md. Shah Amran

**Affiliations:** ^1^Department of Pharmacy, Faculty of Pharmacy, University of Dhaka, Shahbag, Dhaka 1000, Bangladesh; ^2^College of Pharmacy, University of Houston, Houston, USA; ^3^Department of Pharmaceutical Chemistry, Faculty of Pharmacy, University of Dhaka, Shahbag, Dhaka 1000, Bangladesh; ^4^Department of Pharmaceutical Technology, Faculty of Pharmacy, University of Dhaka, Shahbag, Dhaka 1000, Bangladesh; ^5^Department of Science & Technology, Tokushima University Graduate School, Tokushima, Japan; ^6^Molecular Pharmacology Research Center, School of Pharmaceutical Sciences, Wenzhou Medical University, Wenzhou, 325035 Zhejiang, China; ^7^Department of Biochemistry and Molecular Biology, Jagannath University, Dhaka 1100, Bangladesh; ^8^Unit of Bee Research and Honey Production, King Khalid University, Abha 61413, Saudi Arabia; ^9^Department of Biology, College of Arts and Sciences, King Khalid University, Abha 61413, Saudi Arabia; ^10^Department of Biochemistry and Molecular Biology, Trust University, Barishal, Ruiya, Nobogram Road, Barishal 8200, Bangladesh

## Abstract

Cancer has been one of the most dominant causes of mortality globally over the last few decades. In cancer treatment, the selective targeting of tumor cells is indispensable, making it a better replacement for conventional chemotherapies by diminishing their adverse side effects. While designing a drug to be delivered selectively in the target organ, the drug development scientists should focus on various factors such as the type of cancer they are dealing with according to which drug, targeting moieties, and pharmaceutical carriers should be targeted. All published articles have been collected regarding cancer and drug-targeting approaches from well reputed databases including MEDLINE, Embase, Cochrane Library, CENTRAL and ClinicalTrials.gov, Science Direct, PubMed, Scopus, Wiley, and Springer. The articles published between January 2010 and December 2020 were considered. Due to the existence of various mechanisms, it is challenging to choose which one is appropriate for a specific case. Moreover, a combination of more than one approach is often utilized to achieve optimal drug effects. In this review, we have summarized and highlighted central mechanisms of how the targeted drug delivery system works in the specific diseased microenvironment, along with the strategies to make an approach more effective. We have also included some pictorial illustrations to have a precise idea about different types of drug targeting. The core contribution of this work includes providing a cancer drug development scientist with a broad preliminary idea to choose the appropriate approach among the various targeted drug delivery mechanisms. Also, the study will contribute to improving anticancer treatment approaches by providing a pathway for lesser side effects observed in conventional chemotherapeutic techniques.

## 1. Introduction

**Figure 1:**
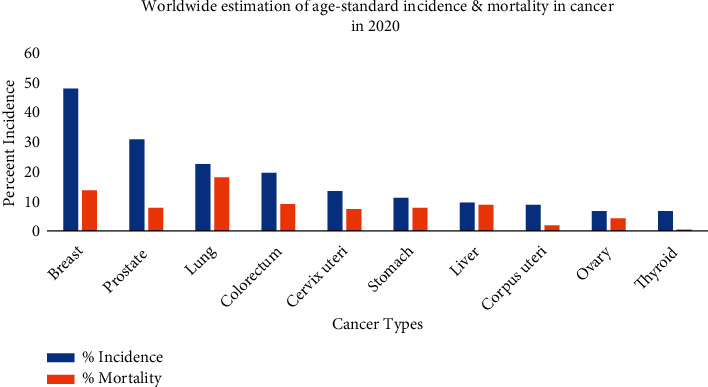
Worldwide estimation of age-standard incidence and mortality in cancer in 2020 (redrawn from [2]).

“Wir müssen chemisch zielen lernen” a German phrase that means “we have to learn how to aim chemically” is a postulate originated by Paul Ehrlich, the founder of chemotherapy. He was awarded the Nobel Prize for Physiology and Medicine [1]. Cancer occurrence is rapidly increasing worldwide and is anticipated to rank the leading cause of death (Figure 1).

Cancer is the result of aggressive dividing of cells by several mutations that subsequently compete with normal cell for nutrients and then form a bulk mass. At one point, angiogenesis occurs when tumors create vasculature of their own, which leads to a hostile microenvironment reflecting increased interstitial pressure (IFP), oxygen and nutrients deficiency, severe acidity, and so on [3]. Due to the distinct features of the tumor microenvironment's ability to activate immune checkpoint molecules and produce a variety of immunosuppressive cytokines, the tumor can evade the immune system. Tumor cells produce cytokines and secrete growth factors and extracellular matrix in order to develop by changing the milieu around them. In turn, this inhibits the immunological response [4]. However, the intricate tumor microenvironment offers the benefit of targeting drugs at the cancer site [3]. In oncology, a conventional drug delivery system results in the death of promptly dividing and growing cancer cells by opposing the mitosis phase of a cell cycle and interfering with DNA synthesis [5]. Radiation and chemotherapy have harmful side effects and toxicity that lower the quality of life. Additionally, they carry the danger of making cancer cells resistant to these therapies [6]. Nontargeted chemotherapeutic agents lead to numerous disastrous unpremeditated and adverse effects by damaging healthy, more specifically rapidly growing healthy tissues, such as bone marrow cells involved in blood production, hair follicles, and cells inside the mouth cavity, cells that are present in the gastrointestinal tract and reproductive system. Cognitive impairments; immunosuppression; bone marrow suppression; gastrointestinal discomfort; fatigue; hair loss; organ damage; infertility; anemia, secondary tumors; urine and bladder changes and kidney problems; sores and pain with swallowing due to problems associated with mouth, tongue and throat; unexplained bruising and bleeding; sensitivity to infection; dry and pale skins; and blood stools are the common toxicities associated with conventional chemotherapeutic agents as these pose nonselective action to normal cells and are given to cancer survivors at a maximally tolerated dose (MTD) to approach maximum tumor cell death resulting in suboptimal treatment due to excessive toxicities. Conventional chemotherapeutics' failure to acquire multidrug resistance (MDR), and nontargeted toxicity can only augment progression-free survival in cancer patients but seriously decreases their quality of life and even results in death. A British inquiry, by The National Confidential Enquiry into Patient Outcome and Death (NCEPOD), after the investigation of more than six hundred mortality cases within thirty days of chemotherapy, presented that 43% of patients suffering from significant toxicity due to treatment and 25% of patients' death instigated from the negative side effects of chemotherapy itself rather than the malignancy. The study demonstrated causation or augmentation of 27% of death in severely ill patients receiving chemotherapy [7].

These drawbacks can be dealt with if drug preparations are delivered through different mechanisms that involve selective and quantitative accretion of drugs independent of site and administration methods. For this purpose, nanotechnology has been developed for drug delivery selectively to the diseased microenvironment with additional improvement in the bioavailability and solubility properties, altered bio-distribution of chemotherapeutics, and alleviation of drug resistance due to long-term treatment [8, 9]. A targeted drug delivery system can provide a platform where those associated unintended side effects and toxicities in normal cells can be reduced by enhanced drug accumulation and increased intracellular concentration of drug in a cancer cell as well as decreased drug efflux from cancer cell [10]. In several studies, active targeting nanocarriers have shown to be more expeditious in enhancing drug accumulation in cancer cells; thus, providing a massive role in modern cancer chemotherapy as well as in cancer therapy with herbal and traditional medicines to improve efficacy and safety profile [11]. A broad spectrum of studies related to different targeting approaches has been performed and is still going on. So, this is necessary to bring out the core approaches to have an overall idea regarding the targeting mechanism. As of now, no broad-spectrum discussions have been performed in this area. We have summarized the major targeting mechanisms, EPR (enhanced permeability and retention), stimuli-, chemical-, and receptor-based targeting, with the help of diagrams and tables. The article concentrates only on these four basic drug targeting methods because of their potentiality over other methods like inverse, dual, and double targeting. The inverse targeting is limited to targeting drugs to non-RES organs only. The dual and double targeting approaches cannot be considered in basic type of drug targeting because these strategies can be incorporated in the primary targeting methods mentioned in this article to enhance the efficacy of drug trafficking and accumulation to target [12]. Due to the EPR effect and the absence of lymphatic drainage, the concentration of nanodrugs gradually increases in tumors, eventually reaching levels that are many times higher than those in plasma. The discovery of the EPR effect was a significant development in drug delivery systems, and from its origin, there has been tremendous anticipation for using this effect to administer selected anticancer drugs. [13]. On the other hand, enhancing selectivity, biocompatibility, cancer microenvironment-based sensitivity, and clinical acceptance are the crucial characteristics of stimuli-based drug delivery systems. Drug release at the target site may be expedited or triggered, cellular binding and internalization may be improved, or drug perfusion may be more efficient across the tumor volume when pharmaceuticals are delivered via stimuli-responsive carriers, lipids, and/or prodrugs in the tumor milieu. Multiple stimuli-responsive drug delivery systems that can increase tumor necrosis by a number of folds have been developed in the modern period [14]. Chemical-based targeting allows the application of prodrug and double prodrug-basedsite-specific targeting [15]. Biological receptor-based targeting involves the use of one or more targeting moieties attached to the nanoparticle surface that particularly interact with antigens or receptors that are either expressed differently or excessively on tumor cells in comparison to normal tissues [16]. This article summarizes different aspects of the aforementioned targeted drug delivery approaches that may help researchers in cancer drug design and development with a broad-preliminary idea of different ideas of the targeting methodologies, their drawbacks, and how to overcome them. The future opportunities have also been discussed that may provide assistance to further improve the site-specific drug delivery approaches.

## 2. Site-Specific Drug Targeting in Different Cancer Treatments

It was reported that the anticancer medication doxorubicin (DOX), which is enclosed in a PEGylated liposome, was successfully delivered to the estrogen receptor (ER) for the treatment of breast cancer. To target ERs, estrone (ES) was attached as a ligand to a stealth liposome (ES-SL-DOX). ES-SLDOX was accumulated in the tumor tissue at rates that were 24.27 and 6.04 times greater than those of free DOX and SL-DOX, respectively. These results suggest that the estrogen receptor(s) may be used as a possible target for cancer treatment, and estrone-anchored stealth liposomes may be one of the promising approaches for the side-effect-free delivery of anticancer agents to breast tumors [17]. In a different study, estrone was attached on pH-sensitive liposome surfaces to enable drug targeting to ERs. When the pH was acidic, the estrone-anchoredpH-sensitive liposomes (ES-pH-sensitive-SL) displayed fusogenic potential (5.5). Studies on the in vitro cytotoxicity of ES-pH-sensitive-SL formulation on ER-positive MCF-7 breast cancer cells showed that it was more cytotoxic than non-pH-sensitive targeted liposomes (ES-SL) [18]. Surface modification can improve the targeting potential of the nanoparticles. A study was conducted that shows when chitosan/poly (ethylene glycol) (CTS/PEG) nanoparticle was conjugated with anisamide to create CTS/PEG-AA, a higher antitumor activity and less cytotoxicity were observed. This opens new avenues to treat lung cancer with Gemcitabine with few side effects and increased efficacy [19]. Greater molecular deposition in tumor site can be achieved by utilization of the two or more targeting strategies at a time. A study with magnetic aerosol drug targeting in lung cancer therapy using permanent magnet revealed that in passive targeting, a small amount of particle deposition occurs on the lung branches. However, the addition of a permanent magnet close to the tumor increased the particle deposition fraction for the 7 m-diameter particles by up to 49%. Additionally, the magnetic size optimization can increase particle deposition by 68% [19].

Prostate cancer (PC) is the second most common cancer in men diagnosed worldwide (903 000 new cases, or 13.6% of the total) and the fifth most prevalent cancer overall. PC develops into a castration-resistant (CR) state over time, which is still incurable. There is an urgent need for the creation of novel targeted therapeutics due to the lack of effective treatments for PC. PSMA is a type II integral membrane glycoprotein [20] that is frequently utilized as a PC cell marker and a well-known imaging biomarker for therapy monitoring. The majority of prostate cancers, including undifferentiated, metastatic, and castration-resistant PC, are linked to its enhanced expression. PMSA is a highly desirable target for antibody-based diagnostic and therapeutic treatments in PC because of its highly limited expression in the prostate and overexpression in all prostate carcinoma stages [21]. Nutlin-3a, a potentially effective anticancer medication, has a number of drawbacks, including low solubility, nonspecific delivery, toxicity, short circulation times in tumor tissue, efflux by transmembranous proteins, and knockout by cellular lysosomes, which reduce the effectiveness of the drug's response [22]. The aforesaid restrictions can potentially be overcome by nanotechnology, allowing for the best possible application of this effective medicine in regaining p53's ability to suppress tumors and treat cancer. A potential carrier for medication loading, targeting, and release to the target cell for treating CC is a nanoformulation of Nutlin-3a coupled to a targeting epithelial cell adhesion molecule and enclosed in PLGA NPs [23].

## 3. EPR (Enhanced Permeability and Retention)-Based Drug Targeting

A universal pathophysiological phenomenon and mechanism known as the “enhanced permeability and retention effect” (EPR effect) explains how certain macromolecular substances, such as albumin and other polymer-conjugated drugs, can gradually accumulate in the tumor vascularized area to target the delivery and retention of anticancer drugs into solid tumor tissue. In this case, nanoparticles are designed in an optimum size that allows their penetration deep into the tumor vasculature due to its complex nature [24].

In 1986, it was found that after intravenous injection of plasma albumin binding Evans blue, a selective dye was accumulated in tumor tissues. A similar case was observed for 90 kDa transferrin and immunoglobulin G, having a molecular weight of around 160 kDa. But opposite behavior was observed in terms of neocarzinostatins and ovomucoid having molecular weights of 12 kDa and 29 kDa, respectively [25]. It is confirmed that polymeric drugs that cross the molecular weight required to pass through the renal tubule accumulate in tumor tissue for an extended time after being given intravenous injection based on which nanosized drugs are being developed [26].

**Figure 2:**
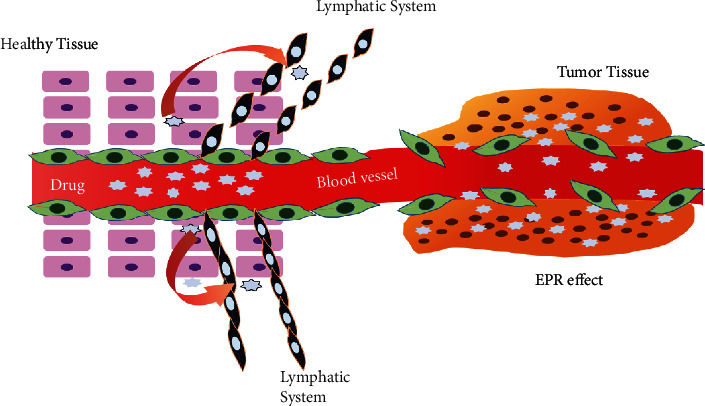
EPR effect in tumor (redrawn from [27]).

As illustrated in Figure 2, during tumor cell multiplication, angiogenesis-induced neovasculature is formed [28–31], which has some distinctive properties to normal cells: dilated, leaky, irregularities in shape [32]; unaligned endothelial cells with abnormal/nonappearance of perivascular cells [22] leading to defective production of basement membrane, smidgen drainage from lymphatics [33, 34]; and absence of vascular smooth muscle cell which is responsible for the appropriate response of chemical mediators such as acetylcholine, adrenalin, nitric oxide (NO), bradykinin (BK), adrenalin, and calcium which sustains a constant blood flow and volume by balancing vasoconstriction and vasodilatation effect [26]. Besides, the vasculature characteristics are altered as well: relatively wide lumen, impaired angiotensin II receptors [33–38], which results in vasodilation and subsequent increase in extravasation, and increased vascular pore size (human colon carcinoma showed a pore size of 400 nm) [36]. All these phenomena contribute to the tumor's increased permeability and retention effect. Moreover, factors that facilitate permeation through vasculature are vascular endothelial growth factor (VEGF/VPG) [39, 40], nitric oxide [41, 42], bradykinin [43, 44], peroxynitrite (ONOO-), matrix metalloproteinases (MMPs) [45], prostaglandins (PGs) [46], and cytokines-like tumor.

Necrosis factor-*α* [47] are released that facilitate the EPR effect. Restricted lymphatic drainage at the tissue sites of tumor vasculature results in accumulation and retention of drug molecules [26]. In MCa-IV mouse mammary carcinomas, the mechanism of tumor vasculature leakiness was examined using complementary light and electron microscopy that showed defective endothelial monolayer, endothelial fenestrae, abnormal shape of the cells, and cells being overlapped or loosely interconnected [35].

In contrast with macromolecular ones, drugs with small molecular mass can easily get leaked from a blood vessel and distributed in tissues, which accounts for the drug to be distributed in normal healthy tissues and tumor-affected tissues. The associated toxicity limits the use of small molecules in drug targeting [48]. Moreover, the half-life of high molecular weight drugs is augmented mainly due to slow venous return from interstitial space, poor excretion through the kidney, and the reticuloendothelial system's capability to remain unrecognized by the reticuloendothelial system. Lack of functional lymphatics is a characteristic feature of tumor tissues. The macromolecules are mainly drained through this lymphatic system because the venous return is significantly slowed down in tumor tissues. Consequently, poor lymphatic drainage contributes to the pronounced accumulation of nanosized drug particles in the tumor microenvironment. On the other hand, in both tumor and healthy tissues, small molecular weight agents can freely move out from the interstitial space to the venous end of the capillaries hindering their retention capacity in the tumor [26, 49]. There is no size of drug product yet specified for achieving the desired EPR effect. Macromolecules with a size of 10 to 200 nm [50]/100 to 400 nm [51]/100 to 200 nm [50] have been claimed in different studies for achieving optimum effects in drug targeting through effect. Molecules with smaller weights than ca. 40000 tend to have a diameter of approximately 5 nm and show pronounced excretion through the kidney's filtration system [52]. Also, the upper limit of the size assists in camouflaging the drug from the reticuloendothelial system, which scavenges carrier systems more prominent than 400 nm through a nonspecific mechanism [53]. In a study with mesoporous silica nanoparticles (MPSNPs), it has been shown that the nanoparticle diameter must be >10 nm to prevent kidney clearance, but <400 nm for effective diffusion into the tumor and accumulation in tumor tissues through an enhanced EPR effect [54]. However, nanoparticles with a diameter of 100 to 300 nm have distinctive physiological characteristics. Additionally, nanoparticles smaller than 50 nm are dispersed randomly throughout the body as a result of their unfettered migration through fenestrated blood arteries [54]. Therefore, the ideal size range for MPSNPs is between 50 and 300 nm for greater cellular absorption, better tumor accumulation, and prolonged circulation time. Nude mice were used to test the functionalized MPSNPs with various sizes on the doxorubicin-sensitive HeLa squamous cancer cell lines (KB-31) [54].

However, EPR-based methods are subjected to some challenges. Primarily, enlarged, convoluted, and haphazard channels lead the blood to flow heterogeneously within tumor tissue. Along with this, hypoxic and acidic intratumor conditions resist nanosized drugs to act against the progression and metastasis of tumor by preventing the drug from penetrating deep into the tumor (Huynh and Zheng, 2015; Ziyad and Iruela-Arispe, 2012). Additionally, continuous extravasation of plasma proteins and other substances contributes to higher interstitial fluid pressure (IFP). In a tumor, interstitial fluid pressure increases from 10 to 40 mm Hg compared to normal tissues, which hinders the delivery of drugs to the target adequately. Furthermore, excess cellular multiplication in a particular region leads to solid stress and mechanical force, which compress tumor vessels and reduce perfusion [55–58]. Also, cancer-associated fibroblasts (CAFs) lead to diminished uptake of drugs by upregulating the synthesis of extracellular matrix (ECM), ECM remodelling by matrix metalloproteinase (MMP), post-translational modifications, and several other ECM-mediated malignant changes [59, 60]. Abovementioned factors interrupt the EPR effect for the delivery of drugs to the tumor, which can be overcome by performing some physiology and drug-based actions. It has been observed in research on tumor-bearing rats, 51Cr-albumin or neocarzinostatin (NCS) conjugated with 51Cr-labeled styrene-co-maleic-acid polymer (SMA), which is called SMANCS, when injected, 1.3-3 fold intensification of drug accumulation was perceived after systolic blood pressure was increased from 100 to 150 mm Hg using angiotensin-II [61]. Because of vasoconstriction and tighter endothelial junctions, the bone marrow, kidney, and other healthy organs were deficient in drug distribution. The mechanism of such intervention is that vasoconstrictors constrict healthy blood vessels but not the tumor vessel due to the abnormal muscular organization where a comparative increase in blood flow is observed. Nonetheless, this process is limited by the demographics of people having hypertension [62]. Vasodilators like isosorbide dinitrate that releases NO can enlarge the endothelial gap of arteries associated with tumors [63, 64]. Also, ACE inhibitors prevent the degradation of bradykinins in vivo and enhance their accumulation [65–67]. Some more examples of agents that increase permeation of drug through vasodilator include carbon monoxide-releasing micelles [68, 69], PGs like PGE1 and PGI2 [66], and botulinum neurotoxin type A [70]. Anthracyclines, nitrosourea, mitomycin C, and SMANCS are proinflammatory anticancer agents that can induce vascular permeability and, thereby, a pro-EPR effect by generating agents like superoxide NO activating pro-MMP to MMP and in many other ways [64, 71, 72]. For the induction of a stable and inert cavitation effect, a combination of ultrasound and microbubbles can be used. This results in the opening of the tight junctions and consequently the permeation. However, the drawback of this method is that nonspecific targeting may also harm the normal healthy tissues [73–75]. Vascular endothelial growth factor (VEGF) and platelet-derived growth factor (PDGF) can modulate subendothelial structures and enhance the extravasation of macromolecules. But due to the presence of receptors on both endothelial cells and tumor cells, the use of growth factors is limited [76–78]. TNF is said to cause hemorrhagic necrosis in mice. Likewise, studies were conducted on patients with melanoma or sarcoma of the extremities. It shows a shred of clear evidence a high dose of TNF-*α* can promote the delivery of chemotherapeutic drugs. However, the drug has a very high toxicity profile and narrow therapeutic index, causing various side effects systemically in humans [79–84]. Studies showed that a relative increase in the extravasation of 100 nm liposome was observed in the case of the tumor but not with normal vasculature when hyperthermia was induced [85]. Also, murine mammary carcinomas were used to demonstrate the significance of hyperthermia in tumor-specific drug delivery [20]. Another study regarding local hyperthermia showed that the extravasation of tumor vasculature was observable up to 10 *μ*m diameter of liposomes [20, 86, 87]. Elevated IFP is the outcome of angiogenesis and drugs like Imatinib [88], Paclitaxel [89], Bevacizumab [89], Combretastatin by disrupting vasculature [90], and ZD6126 is a tubulin-binding agent [91] and hence been shown to reduce IFP, admittedly allowing penetration of the drug. An oral DNA vaccine proposed by a group of scientists' targets fibroblast activation protein (FAP) and then kills CAFs, mediated via CD8+ cells by the suppression of tumor cell progression along with the metastasis of murine colon and breast malignancy. Because of the death of CAFs, ECM production and deregulation get halted, leading to the inhibition of collagen synthesis [60]. Besides, anti-TGF-*β* antibodies [92], Losartan [93, 94], and Pentoxifylline [95] have been proved to have the antifibrotic effect which reduces tumor growth by promoting the uptake of chemotherapeutic drugs. A study by a group of scientists reported paclitaxel could cause alteration of the condensed tumor cell and expand the interstitial space, improving penetration of drugs like doxorubicin-HCl liposomes in a schedule dependent manner [96]. In radiation-treated tumors, a 2.2-fold higher increase in the rate of entry than nonirradiated tissue of nanosized molecules was observed, although radiation may damage the vessels, negatively affecting nanodrug delivery [97]. A study found that compared with the control tumors, photodynamic therapy and light therapy can improve the EPR effect almost up to three times [97–99]. PEGylated gold nanorod conjugated with GRP78-targeting peptide and subsequent light application increased the EPR effect 2-fold compared with untreated controls. Apart from this, improved drug delivery was observed upon the administration of radio immune conjugates [100–102]. In this process, antibody (targeted mab) photo-absorber conjugate (APC) binds to targeted tumor molecules, improving vascular permeability by inducing perivascular cell death. The increased permeability permits nanosized particles surprisingly to pass in the tumor beds, called super-enhanced permeability and retention (SUPR) demonstrated twenty-four fold intensification in nanodrug transport compared with the tumor not treated [101, 103]. A stimuli-responsive drug delivery system can take advantage of endogenous and exogenous stimuli. Endogenous stimuli include increased interstitial H^+^ ion concentration; overproduction of several enzymes like matrix metalloproteinases, phospholipases, and glucose-oxidases; and a higher concentration of glutathione, redox gradient, etc., be employed for selectively triggering the nanoparticles to promote the release of the transported drug. External stimulation such as temperature fluctuations, light and electric fields, application of variable magnetic fields, and high energy radiations, enhances a carrier's activity and further triggers drug release at the disease site to assess disease identification and/or preferred effect of this therapy (Hatakeyama, 2017; Mi, 2020).

## 4. Stimuli-Based Drug Targeting

The term “stimuli responsive materials” or “environmentally responsive materials” refers to a phenomenon that influences an activity at a specific site or target tissue to bring about useful activities for the release of the drug via various mechanisms. Materials that alter physically or chemically in reaction to an external stimulus are said to be stimuli-responsive materials. Due to their biomimetic origin, these materials demonstrate the environment responsive behavior phenomena and react to outside stimuli [14].

### 4.1. Exogenous Stimuli

Some external stimuli-based approaches for enhancing drug delivery towards a target are delineated and summarized in Table 1.

#### 4.1.1. Temperature-Responsive Drug Targeting

**Figure 3:**
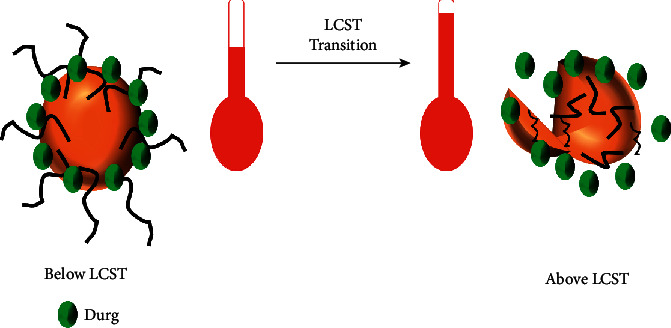
Temperature-sensitive drug release (redrawn from [125]).

**Figure 4:**
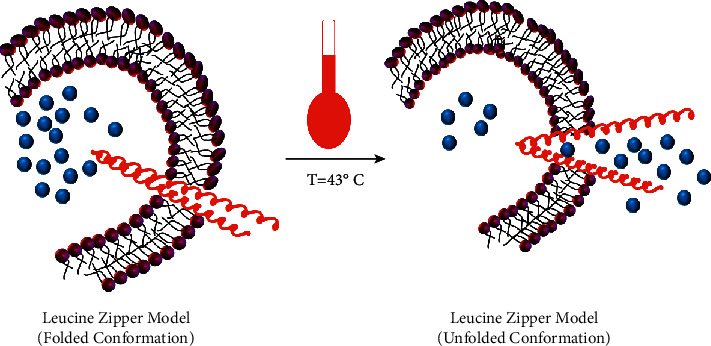
Leucine zipper model in thermosensitive drug delivery (redrawn from [121]).

The concept of applying hyperthermia for the delivery of drugs to specific diseased regions was first proposed at the end of the 1970s, which involved the utilization of thermo-labile liposomes [121]. Using the idea of lower critical solution temperature by polymeric micelles or liposomes or nanocarriers (usually poly(N-isopropyl acrylamide) (PNIPAM)) [107, 122], leucine zipper peptide-liposome hybrids [123, 124], and bubble-generating liposomal system are some of the strategies which utilize the sensitivity towards temperature for drug delivery to the specific microenvironment. For thermosensitive polymer micelles formation, the preferable polymer building block is PNIPAM which exhibits lower critical solution temperature and induces phase transition of the lipid elements and bilayers' conformational changes. Thermo-labile polymeric materials could move towards nonpolar from polar state to generate nanocarriers which undergo selfassembly at elevated temperature (above 32 Celsius) than LCST and, in contrast, get disassociated from each other when temperature drops (Figure 3) [107, 126]. Through the oscillations of frequencies in the range of radio waves, controlling of water bags by temperature or employing microwave activation through the miniature annular-phased array, heat is generated exogenously [121]. Additional polymeric constituents, for example, poly (*γ*-2-(2-(2-methoxy ethoxy)-ethoxy) ethoxy*ε*-caprolactone)-b-poly (*γ*-octyloxy-*ε*-caprolactone) also have increased the discharge of drug at low hyperthermia (40°C) [127] as their property and structure can be altered to ensure the temperature for transition is nearby the temperature of the body and thus providing convenience for site-specific such as subcutaneous or intratumoral or peritumoral administration. Leucine zipper peptide–liposome hybrids utilize a peptide, as shown in Figure 4, that leads to drug release through the transformation from its unfolded conformation to the folded one by the application of heat [124].

Thermoresponsive bubble-generating liposomal is a promising system where ammonium bicarbonate forms carbon dioxide at mild hyperthermia (42°C), creating a permeable pore through the lipid bilayer [104]. Moreover, local hyperthermia can induce an on-off regulator of the function of cell-penetrating polypeptides like diblock-copolymer elastin, which can penetrate the cell through the control of the on-off mechanism induced by local hyperthermia. Assembly of such peptides leads to greater than eight times uptake of HeLa-cells [128]. Cryotherapy or cold shock is also a thermosensitive technique for the diffusion of encapsulated drugs where reduction of temperature results in increased porosity due to swelling or deswelling of nanocarrier like pluronic F127–polyethyleneimine [106].

#### 4.1.2. Magnetic-Responsive Drug Targeting

**Figure 5:**
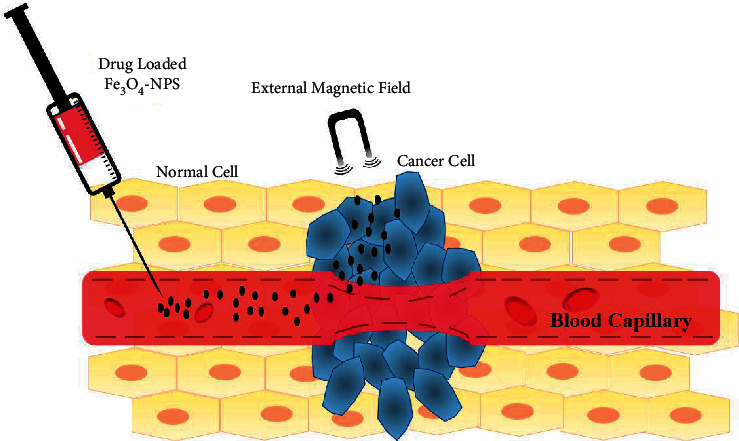
Magnetic responsive drug delivery (redrawn from [133]).

In tumors located in high-risk healthy tissues like the brain where immediate surgery is not indicated due to the risk of haemorrhage and tissue injury, magnetically responsive nanocarriers show promising effect in tumor targeting through intrinsic tropism along with the generation of local hyperthermia to promote drug release by implying alternating magnetic field (AMF). Magnetic tropism can be achieved for the injected magnetically sensitive nanocarriers by introducing an extracorporeal magnetic field on the disease site. Magnetic guidance is coupled with local hyperthermia that may result from hysteresis loss and/or Néel relaxation [121]. Therefore, the synergistic cytotoxic effect can be achieved by combining thermosensitive polymers and lipids as a coating material for magnetic nanoparticles (e.g., crosslinked PNIPAM hydrogels loaded with Fe_3_O_4_ nanoparticles) that has been observed in tumor treatment where thermosensitive cleavage releasing heat shock protein inhibitors (i.e., geldanamycin) has been used to block the antiapoptotic protein leading to resistance-free apoptosis [129, 130]. The on-off states of AMF can moderate drug release control by the contraction of the size of the mesh and repossession of the gel. Additionally, AMF can contribute to structural alteration, for instance, augmented porosity of shell or bilayer, breakdown of magnetic nanoparticle core, or deformation of single-crystal nanoshell lattice [131, 132]. However, the magnetic direction is not a promising strategy because this is limited to the tumor nodules accessible for treating metastatic or disseminated tumors. Besides, the external magnetic field involves complexity applying sufficient strength to penetrate the depth of tissues targeted at the diseased region since its setting up requires adequate attention and deep penetration into the tissues (Figure 5) [121].

#### 4.1.3. Ultra Sound-Responsive Drug Targeting

**Figure 6:**
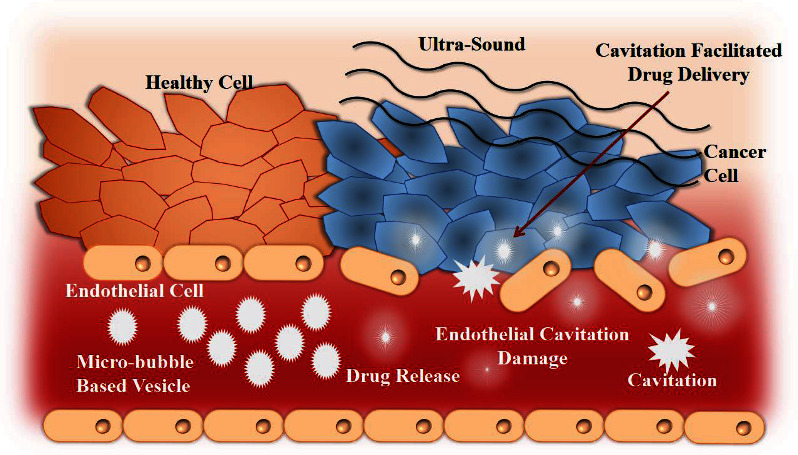
Ultrasound responsive drug delivery (redrawn from [135]).

Ultrasound stimuli can be effective in diagnosis, imaging, and drug delivery due to their noninvasive nature and high safety. The intensity of ultrasound can be adjusted according to different desired applications. Less than 20 kHz frequencies can be applied for imaging, while upper than that are used for disrupting nanocarriers and enhancing cancel cell membrane permeability [134]. Various nanocarriers, such as microbubbles, can trigger the delivery of drugs by implementing thermal or radiation forces or mechanical effects produced by cavitation that destabilize nanocarriers, followed by drug release. Although the cavitation threshold leads to a transient increase in vessel permeability, this can also cause metastatic propagation. Therefore, several ultrasound contrast agents and microbubbles are utilized at frequencies required for diagnosis to lower the cavitation initiation point. The use of microbubbles is constrained due to their short lifecycle, large size (1-10 um), and absence of extravasation that can be overcome by perfluorocarbon (PFC) nanoemulsion application of therapeutic frequencies that get transformed into microbubbles. The formation of bubbles through acoustic globule vaporization and subsequent cavitation promotes intracellular penetration and/or release of drugs in the tumor site entrapped in nanocarriers (Figure 6) [136].

Additionally, ultrasound can disrupt vascular integrity, thereby increasing the gap in tumor vasculature [137–139]. Another promising ultrasound drug delivery approach applied in DNA transfection includes extravasation of the drug across the plasma membrane to direct cytosol with the help of aperture formation and subsequently sidestepping the endocytotic pathway that can bring about enzymatic degradation to the drug [140].

#### 4.1.4. Light Stimuli-Responsive Drug Targeting

**Figure 7:**
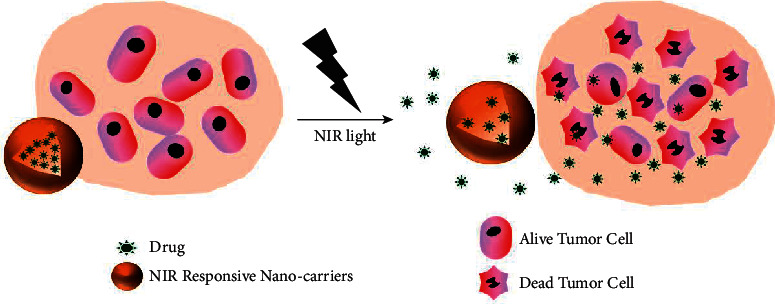
Photosensitive drug delivery (redrawn from [142]).

Light-sensitive drug delivery can be triggered by UV-Visible (<700 nm) or near-infrared (NIR) light (700-1,000 nm) range that provides the advantage of adjusting the radiation wavelength to be applied according to necessity as well as modifying the strength and region of application based on the diseased site morphology to minimize potential harm to healthy surrounding tissues [141]. Light-sensitive nanocarriers can respond to light, as depicted in Figure 7, and perform activities such as conformational change of molecules [143], nanocarriers disassociation due to cleavage of light-sensitive chemical bonds [144], triggered release of therapeutics from nanocarriers in the diseased microenvironment [145], photoacoustic imaging [146, 147], and generating reactive oxygen species for photodynamic and photothermal therapy [148–151]. The photo-switching function of nanocarriers can not only be applied for loading and subsequent UV-vis triggered release of anticancer bioactive compounds, including paclitaxel, doxorubicin, and doxorubicin etc., [151] but also to induce light reversible aggregation of nanoparticles [152]. Nevertheless, UV-visible light is limited because of its short wavelength having low penetration depth (∼10 nm), which allows drug delivery only to directly illuminated areas like the eye or the skin. NIR light-sensitive nanocarriers can overcome this drawback by ensuring the permeation of drugs into more distant tissues from a blood vessel, intrinsic lesser scattering properties, and the lowest possible damage to healthy tissues [153]. Photosensitive-induced structural modification, for example, reversible photo-isomerization of the azobenzene group and compounds produced from their improvement, can be employed for the photo-regulated mechanism of drug release [123, 154, 155]. The light-triggered drug delivery can be applied in transferring genes into the cytoplasm, allowing the genes to avoid the degradation in endosome/lysosome [116].

### 4.2. Endogenous Stimuli-Based Drug Targeting

**Figure 8:**
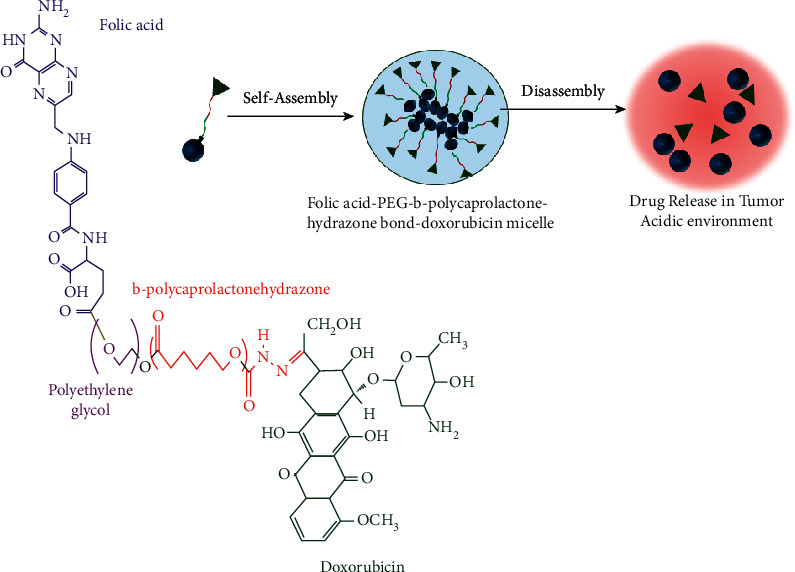
pH-sensitive drug delivery (redrawn from [170]).

Different endogenous stimuli-based approaches have been utilized for cancer-specific drug delivery that are summarized in Table 2. The delivery and the release of therapeutics in certain physiologic regions such as the GI tract/vagina or intracellular compartments like lysosomes/endosomes can be controlled utilizing subtle pH changes in the cancer microenvironment. There are some strategies used here. One is pH variation leading to conformational and/or solubility change of polymers containing ionizable groups. Other techniques include the discharge of molecules attached to a polymeric backbone, altering the polymeric charge, or exposing the targeting ligands via the cleavage of acid-sensitive bonds [121]. Generally, the cytosol, blood, and normal healthy tissues have pH values around 7 to 7.4, while the endosome/lysosome exhibits approximately 6 to 4. Pathological sites like tumors demonstrate 6.5 to 6.8 [166], which is the consequence of angiogenesis in rapidly growing tumors leading to nutrient deficiency. It activates the glycolytic metabolic pathway causing the formation of acidic metabolites. Efficient pH-sensitive systems like chitosan respond to the subtle change of H^+^ ion concentration through proton attachment to the amino group (pKa ∼6.3) and subsequent swelling that brings about the liberation of encapsulated tumor necrosis factor-alpha (TNFa) in the tumor sites [167]. Leakage of camptothecin due to the sudden disassembly of PEG-poly (*β*-amino ester) micelles [168] and delivery of protein in ischemic areas with piperidine- and imidazole-modifiedPEG-poly (*β*-amino ester) micelles [169] are some of the examples of pH-sensitive drug release. Another such application of pH responsiveness is illustrated in Figure 8, where doxorubicin is delivered once the micelles undergo receptor-mediated endocytosis and encounter an acidic pH condition leading to the breakdown of hydrazine bond through hydrolysis [170].

Dissolubility, selectivity, and drug action time issues are common with conventional antitumor chemotherapeutics, and it has proven challenging to obtain high antitumor efficacy with single-drug therapy. Currently, two or more medications taken together in combination therapy are frequently used to treat cancer, but this approach has a drawback in that the drugs do not all act simultaneously, which reduces their effectiveness. As a carrier for the anticancer medications gemcitabine (GEM) and paclitaxel (PTX), which can release medicines to the tumor site simultaneously to produce the antitumor effect, a pH-responsive peptide hydrogel was developed by a group of researchers. The result shows, in the medium at pH 5.8 and pH 7.4, PTX was released from the hydrogel at 96.90% and 38.98%, respectively, over the course of 7 days. In medium with pH values of 5.8 and 7.4, 99.99% and 99.63% of GEM, respectively, were released from the OE hydrogel in 3 days. This outcome is due to GEM's high hydrophilicity and ability to diffuse into aqueous solutions fast. The findings show that the peptide hydrogel responds to pH. When the environment is acidic as seen in tumor, it can continually release PTX. The peptide concentration can be adjusted to control the release speed [171].

In another study, a polymeric mixture of agarose, pluronic acid, glutaraldehyde, and methacrylic acid (MAA) was utilized to make the hydrogels. Then, the pH sensitivity of a cross-linked polymeric network for the nonlymphoma Hodgkin's therapy medication cyclophosphamide was measured. It was found that the hydrogels release cyclophosphamide in a pH-dependent. According to the findings, the drug release differs significantly between pH 1.2 and pH 7.4 (p 0.05). At pH 1.2, medication release was at its lowest point, whereas at pH 7.4, it was at its highest point. The MAA monomer's presence in the hydrogels was what caused their pH-dependent behavior. Consequently, in simulated gastric fluid (SGF) at pH 1.2, a greater quantity of protonated COOH groups from MAA reduced ionic repulsion, enhanced hydrogen bonding among the COOH groups, and led the hydrogels to contract. However, in simulated intestinal fluid (SIF), the produced hydrogels' swelling significantly enhanced. This phenomenon could be explained by the fact that in SIF, the osmotic swelling force inside the hydrogel network and the electrostatic repulsion between the carboxylate anions (COO^−^) of MAA cause the hydrogels to expand and swell [172].

Disulfide bonds can be cleaved by glutathione reductase (GSH), most commonly utilized in the cytotoxic release of drugs. In contrast, the diselenide bonds (Se-Se) sensitivity is limited to lower bond energy than the formerly mentioned bonds despite its high redox potential [168, 169, 173]. The concentration of GSH varies in tumor tissues and healthy tissues (intracellular (∼2–10 mM) and extracellular (∼2–10 *μ*M)), which can be utilized in the targeted deliverance of drug from selfassembled amphiphilic copolymers that owns disulfide links within hydrophobic backbone [174] or containing a disulfide bond at the connection of two blocks of polymer [175, 176]. Another scope to utilize redox sensitivity includes the accumulation of reactive oxygen species in inflammatory sites, such as thioketal-mediated carriage of specific TNF*α*–siRNA to the inflammatory regions, resulting in gene silencing after oral administration [177].

In pathological conditions like cancer, inflammation is associated with altered enzyme expression in the extracellular environment of affected tissues, which can be employed in accumulating drugs in the biological target. The utilization of a short peptide sequence for linking polyethylene glycol (PEG) chains and either transcription activator (TAT) functionalized liposomes has been studied recently that can be cleaved by MMP (matrix metalloproteinases) present in the tumor microenvironment and subsequently exposed the bioactive ligands for better intracellular penetration than nanocarriers without cleavable linkers [178]. In an experiment with tumor-bearing mice, it has been observed that 70% gene silencing was attained after the administration of siRNA-loaded nanoparticles systemically [179]. Lysosomal enzyme cathepsin B, which is overexpressed in tumors with malignancy, allows the cargo release from the degradation of polymersomes [180]. The solid tumors are prone to be hypoxic due to poor vascularization that results in cancer advancements such as distant metastasis and locoregional spread [151]. Hypoxia is also associated with acidity brought about by the catabolism of glucose through the glycolytic pathway leading to H^+^ and lactic acid formation [181]. Therefore, this endogenous development of hypoxia allows the targeted delivery of drugs to the tumor or cancer microenvironment with hypoxia-responsive and pH-sensitive nanocarriers [182].

The stimuli-based approaches are faced with many challenges [183]. For example, choosing materials that are secure and sensitive enough to react to small temperature fluctuations around the physiological temperature of 37°C presents a difficulty in the design of thermoresponsive nanodevices [183]. Here, liposomal systems are currently more developed, and they have the most potential for clinical use. In case of magnetic stimuli, the complexity of setting up external magnetic fields, which require optimal focusing and deep penetration into tissues to reach the affected area with sufficient strength, poses a barrier to magnetic guidance. In this regard, efforts are required to determine the optimal magnetic and irradiation technologies [183]. As discussed before, metastatic dissemination is a major drawback in case of ultrasound-based treatment that can be overcome through microbubbles or other ultrasound contrast agents. For light-triggered drug delivery (less than 700 nm), low penetration depth (10 mm), caused by the significant scattering qualities of soft tissues in the ultraviolet-visible region of the spectrum, is the main disadvantage [183]. Therefore, conventional light-induced medication delivery is only effective in areas of the body that can be lighted directly (such as the eye or the skin). However, it is possible to replace the ultraviolet-visible light source with an NIR laser (700-1,000 nm range) with deeper tissue penetration, lower scattering properties, and little damage to tissues by using photosensitive groups that respond to higher wavelengths or utilizing two-photon technology [183].

## 5. Chemical Method

Chemical targeting method utilizes the chemical modification of drug-complex in the tumor microenvironment that allows the drug-conjugate cleavage to release the active drug [15]. The chemical method mainly utilizes prodrug-based control of targeted drug delivery that can be employed in two ways. The first one involves site-specific transport of inactive drugs converted into an active form by the enzymes present in the tissue. In contrast, the second one implicates target site-specific activation of drugs administered systemically [184].

A powerful combination approach of chemical targeting that has shown to increase the lifespan of tumor-bearing mice by 40-60% compared to control involves the use of a molecular chimera attached with a regulatory DNA sequence of transcription which can selectively be stimulated in mammalian cells and a *β*-lactamase enzyme encoding sequence linked to the regulatory DNA. The chimera was anchored with a prodrug form of methotrexate and 5-fluorouracil, which gets converted to its active state after the DNA coding region and *β*-lactamase are expressed at the target site [15].

**Figure 9:**
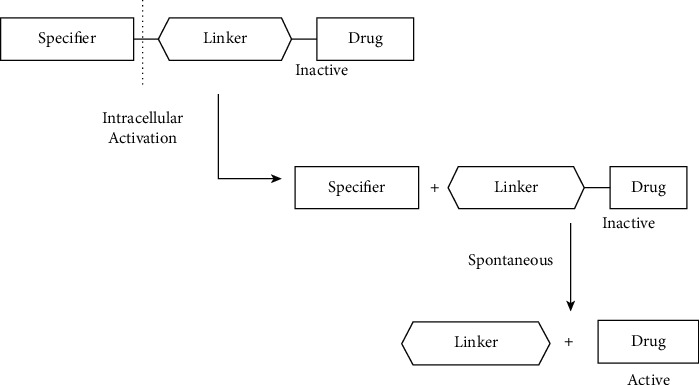
Double prodrug-based approach (redrawn from [187]).

**Figure 10:**
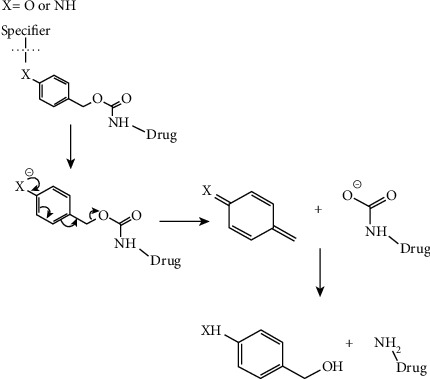
Benzyl elimination: an example of a double prodrug-based approach. It was redrawn from [187].

The use of “double prodrug” provides a solution to the common problem associated with the prodrug approach: the shortage of available chemical approaches that arise from adequate stability issues of linkers in the extracellular environment with a rapid cleavage inside the cell. Due to this phenomenon, the cleavage site must be in correspondence with the chemical bond between the linker and the active drug [185]. This approach involves the addition of stimuli-sensitive moiety called specifier with the linker to attach the active drug, which diminishes the steric impact of the linker on the drug and provides the opportunities for more specifier chemistries than simple prodrug [186]. Figures 9 and 10 give a pictorial representation of the double prodrug-based approach.

For systemic delivery, the drug should be water-soluble. Contrastingly, the drugs should have sufficient lipid solubility to cross cell membranes. An interesting chemical approach was developed by a group of scientists, which allowed the fine-tuning of solubility of a compound at the target site. A water-soluble peptide was attached with a nonpolar drug with the help of a protease-sensitive linker, providing the whole complex water solubility. Based on the specificity of targeted protease for the linker and the ratio of protease activity at the target site, the linker gets cleaved, releasing the lipid-soluble drug. The invention claims to design different linkers targeted by several proteases, for instance, human interstitial collagenase that is active in stomach carcinoma, squamous cell carcinoma of the lung, pancreatic cancer, adenocarcinoma and malignant melanoma, Cathepsin D secreted in breast cancer, and human collagenase type III in carcinoma of the kidney, liver, bladder, colon breast, ovaries, pancreas, lungs, and stomach [15].

A common challenge in chemical targeting involves cleavage of the bonds between the active drug and the conjugate. The tumor site must have the properties to breakdown the drug-conjugate bond. For example, when a drug (specifically paclitaxel) was attached with cis-docohexaenoic acid (DHA), it was discovered that the combination has reduced activity against many cell lines that would typically be amenable to paclitaxel treatment because the paclitaxel in the DHA conjugate was not released into the area around the cells [15].

## 6. Biological Receptor/Ligand-Based Drug Targeting

**Figure 11:**
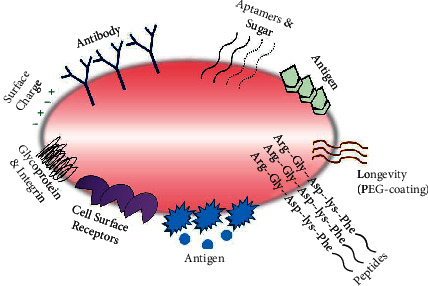
Targeting moieties in cancer cells (redrawn from [189]).

**Figure 12:**
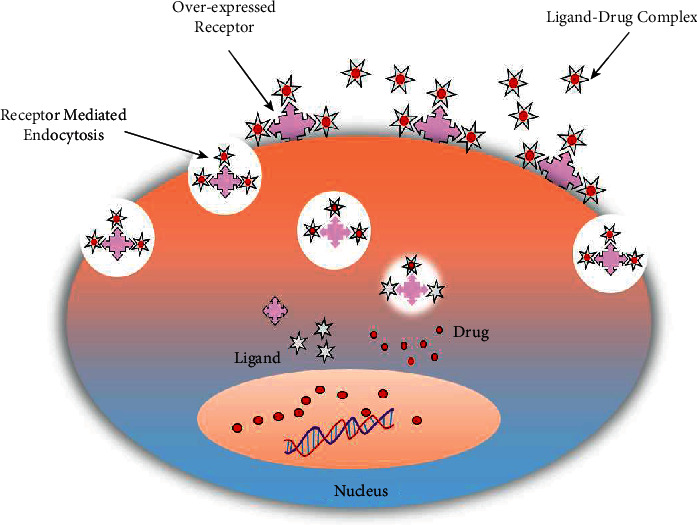
Ligand-based drug targeting (redrawn from [199]).

Ligand-based drug targeting utilizes the overexpressed or uniquely expressed receptor properties on tumor cells to deliver the active drugs. The biological approach focuses on targeting drugs by employing antibodies and fragment, lectin, protein, lipoprotein, hormones, mono/oligo/polysaccharides, ions, and ligands such as folate-based drug delivery systems (Figure 10) [188]. Rakowics-Szolczynska has patented a monoclonal antibody that recognizes a protein found in various carcinomas the in breast, endometrial, cervical, ovarian, and vulval cancers. As illustrated in Figure 11, immunofluorescent detection showed the internalization and localization of the antibody after the attachment with the cancer cell. Gene therapy by attaching genetic materials with these antibodies can be demonstrated either through antisense mechanism or incorporation of the sequence. The proteins showed no detection in normal and healthy tissues, hence representing unique targets for female malignancies [190]. The surface of cancer cells overexpresses various receptors like- transferrin [191, 192], lectin [193], human EGF receptor [194], nuclear [195], lactoferrin [196], folate [197], and integrin [198], which are used for designing target ligands that selectively bind to these receptors (Figure 12).  Table 3 shows some latest receptor-based approaches for drug targeting.

A common drawback associated with receptor-based targeting includes exocytosis due to endosome recycling, for example, exocytosis within 48 hours was observed in the case of active targeting process of hyaluronic acid coated with silica nanoparticle that was internalized first via CD44-receptor. Additionally, conformational alteration of ligands and reduced targeting capability of the nanocarriers in the biological environment due to surface layer formation are some of the common challenges in biological targeting of drugs [16].

## 7. Current Approaches to Fabricate Site-Specific Drug Targeting

Currently, various nanocarriers are being developed with a view to achieving effective site-specific drug targeting. For example, chitosan nanoparticles have mucoadhesive characteristics and are most cytocompetitive when acting in tight epithelial junctions, in contrast to alginate and pectin nanoparticles, which exhibited cytotoxicity in all of the tests. Additionally, a high local drug concentration could lengthen the time of exposure, which would boost the antitumor activity and lessen systemic toxicity in the treatment of cancer, particularly colon cancer. When compared to cationic and neutral polymers, alginate, an anionic mucoadhesive polymer, has a stronger mucoadhesive strength [216]. In targeted drug delivery systems, cellulose and its derivatives are widely used, mostly to alter the solubility and gelation of the therapeutics, which in turn control their release profile. [217]. The drug is released over time due to the hydrogen bonds that existed between the cellulose nanocrystals [216]. Since liposomes' membrane shape is similar to that of cell membranes and they make it easier to incorporate pharmaceuticals into them, they are regarded as superior drug delivery systems. They have also shown to be biocompatible and biodegradable. They also stabilize drug molecules and enhance biodistribution with both hydrophilic and hydrophobic medicines. The medications trapped inside liposomes are not accessible until they are released, which is one aspect of liposomes to draw attention to. In order to maximize medication bioavailability within the therapeutic window at the proper rates and times, as well as in the case of malignancies, this can delay the clearance of lipophilic anticancer treatments [216]. Dendrimers are highly bifurcated, monodisperse, well-defined, and three-dimensional structures which are limited in their clinical applications because of the presence of amine groups. These groups are positively charged or cationic which makes them toxic, hence dendrimers are usually modified in order to reduce this toxicity issue or to eliminate it. Drug loading in dendrimers is performed via the following mechanisms: simple encapsulation, electrostatic interaction, and covalent conjugation [218]. Inorganic nanoparticles such as silver, gold, iron oxide, and silica showed several advantages such as good biocompatibility and versatility when it comes to surface functionalization but still are in the clinical trial stage [216]. Pure solid medicine particles called nanocrystals, which are in the range of 1000 nanometer, have unique properties that enable them to overcome barriers including enhanced saturation solubility, increased dissolution velocity, and increased glueyness to surface/cell membranes. However, this method is rather pricey due to the usage of an organic solvent and its cleanup at the end [216]. Natural biopolymers, which include protein and polysaccharide nanoparticles, are derived from biological sources such plants, animals, microorganisms, and marine sources. The majority of protein-based nanoparticles may be broken down, metabolized, and easily functionalized for attachment to certain drugs and other targeted ligands. Similar to monosaccharides, polysaccharides are made up of sugar molecules joined together by O-glycosidic linkages. The ability of polysaccharides to degrade (oxide) at high temperatures (beyond their melting point), which are frequently required in industrial operations, is one of the main drawbacks of their usage in nanomedicine. Additionally, the majority of polysaccharides are water soluble, which restricts their use in various aspects of nanomedicine, like tissue engineering [219].

## 8. Future Prospects

**Figure 13:**
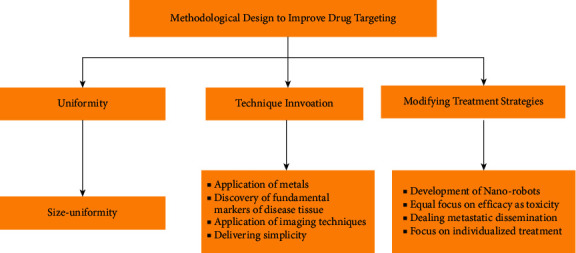
Illustration of the methodological design to improve the drug targeting.

One of the most exciting fields of research nowadays is nanomedicine. Numerous studies conducted in this field over the past 20 years have already resulted in the filing of 1500 patents and the conclusion of numerous clinical trials. There are still some challenges to achieving optimal therapeutic affinity towards the target site. The following contains the methodological design that will help improving the site-specific drug delivery system (Figure 13):

- size-uniformity: the size of nanoparticle for reaching target areas is still not uniform. Some particles are in nanometer while others in submicron level (>100 nm). The next topic of research should be on materials with more uniform consistency and drug loading and release capacity.
- Application of metals: future study into the use of metals, such as gold and silver, for both treatment and diagnosis, has the potential to expand the use of nanomedicines. The gold-nanoparticles that appear to be well absorbed in soft tumor tissues and make the tumor susceptible to radiation-based heat therapy (e.g., in the near infrared region) for selective elimination are a key source of enthusiasm in this approach.
- Discovery of fundamental markers of disease tissue: one important area for future research is the identification of the basic molecular markers of cancer tissues, particularly those that enable absolute targeting without impairing normal cellular function. Therefore, identifying the molecular markers of disease will help progress the use of nanomedicine in the future.
- Development of nanorobots: micro/nanorobots have a lot of potential in medical therapy because they can be used for targeted drug delivery, surgery, cancer diagnostics, and other medical procedures. The engineered micro/nanorobots can move independently, which makes it possible to deliver pharmaceuticals to the hard-to-reach places, unlike standard drug delivery, which depends on blood circulation to reach the target. Micro/nanorobot propulsion is controlled by endogenous or exogenous dynamics, such as magnetic, ultrasonic, and light energy propulsion or chemical/biological reactions. Despite the promising future of micro/nanorobots, the majority of present research is based on in vitro trials; in vivo experiments are s still in the developmental stage. More in vivo biological experiments can make these micro/nanorobots the future of drug targeting.
- Application of imaging techniques: the effect of enhanced permeability and retention is often overrated and/or misinterpreted as scientists do not have sufficient information regarding tumor biology and associated anatomical and pathophysiological characteristics, which is highly heterogenous showing variety from person to person [220, 221]. Among the various ways, one way of addressing the limitation involves the use of contrasting agent labeled nanomedicines with appropriate imaging techniques to monitor the accumulation of tumor and thereby preselect patients [222, 223].
- Delivering simplicity: rational design of formulation is a challenge because it is necessary to take account of size and stability of the formulation which corresponds with the complexity of tumor biology. The addition of linkers that is independent of enzymatic activity for drug release can provide a solution to this problem [224]. A phrase coined by Prestwich can be taken into account which states that we should embrace complexity, engineer versatility, and deliver simplicity [225].
- Equal focus on efficacy as toxicity: in most cases, less attention is given towards improving drugs' efficacy compared to reducing toxicity. Nanomedicines in vast majority of cancers such as multiple myeloma and metastatic breast cancer are only associated with reduced side effects but not much enhanced therapeutic benefit [226]. One way to deal with such problem is to apply external local beam radiotherapy to polymeric nanomedicines synergistically that has exhibited improved accumulation as well as enhanced efficacy and tolerability [227, 228]. Production of VEGF which is a permeability-enhancing growth factor and FGF that causes induction of apoptosis and subsequent reduction of cell density was used to explain the effect of radiotherapy tumor treatment [229, 230].
- Dealing metastatic dissemination: virtually, most of the anticancer medications using nanotechnology are designed for solid locally confined tumors but not for metastatic one which is the major cause of death [231]. Scientists working on the antimetastatic treatment have found different notions, one of which is the development of nanomedicines that have the capacity to modulate immune system, for instance, a human immunoglobulin was developed to evaluate it in four end-stage cancer patients in Czech Republic that presented improved blood parameter evident for lymphocyte-activated killer (LAK) and nuclear killer cell activation [232, 233]. Another possible strategy involves compartmentalized delivery. For example, intraperitoneal injection of cancer-targeted nanoparticles, liposomes, polymers, or micelles showed efficacy in targeting tumor nodules in metastatic ovarian cancer which allowed the drug to be present in the peritoneal cavity where the metastasis occurred. The approach can also be exploited in other locally metastasized carcinoma like liver, pancreatic, or colorectal carcinoma [234].
- Focus on individualized treatment: the approach of treatment strategy varies from person to person depending on the genetic polymorphs, enzymatic expression, and different biological markers. So while drug development, formulation scientists should focus on the individualization.

## 9. Conclusion

The mortality rate due to various types of cancer is increasing every year. Conventional treatment approaches are subjected to various side effects that complicates the life of a cancer-patient or sometimes can lead to death. Therefore, currently, researchers find the site-specific targeting of the molecules as the treatment method for cancer. Enhanced permeability and retention, stimuli-, chemical-, and receptor-based targeting are some of the common and effective targeting approaches that are being exploited by the researchers worldwide. These methods utilize a variety of approaches for drug molecules to reach the tumor microenvironment. Still, there is a huge room of improvements to make these approaches more effective. These approaches are discussed thoroughly here along with their drawbacks and way to overcome those barriers. The future prospect of how to improve these strategies has also been elaborated. The work will provide a broad-window of understanding targeted drug delivery methodologies to the cancer drug development research.

## Figures and Tables

**Table 1:** Examples of different exogenous stimuli-based drug delivery options and different carrier systems along with their application.

Stimulus	Carrier	Associated cargo	Application	Ref
Temperature	Micelles	Nile red doxorubicin	Efficient drug delivery to cancer microenvironments through thermally stimulated drug release	[104]
	Complexes	pDNA	Gene therapy of tumors	[105]
	Nanocapsules	siRNA	In HeLa cancer cells, the intracellular delivery of siRNA has enhanced	[106]
	siRNAsome	Doxorubicin siRNA	Drug action against multidrug resistant cancer	[107]
	Polymersomes	Doxorubicin	Association between thermal and pH-responsive drug release means a dual thermal system.	[108]

Ultrasound	CaCO_3_ nanoparticles	Doxorubicin	Ultrasound imaging of tumor, drug release, and tumor therapy	[109]
	Liposome	Doxorubicin	Cancer diagnosis and light- and temperature-based chemotherapy	[110]

Magnetic	Mesoporous iron oxide nanoparticles	Perfluorohexane and Fe_3_O_4_/Fe_2_O_3_ nanoparticles, and paclitaxel	Thermalchemotherapy aiming active tumor	[111]
	Polymeric micelles	La0.7Sr0.3MnO_3_ and doxorubicin	Effective breast cancer theranostics	[112]
	Magnetic nanoparticles	Fe_3_O_4_/Fe_2_O_3_ nanoparticles	Treatment of primary along with metastatic lung carcinoma	[113]
	Nanoparticles	Fe_3_O_4_/Fe_2_O_3_ nanoparticles doxorubicin	Chemotherapy and hyperthermia induction through the magnet to treat active tumors.	[114]
	Porous magnetic microspheres	Fe_3_O_4_/Fe_2_O_3_ nanoparticles and perfluorohexane	Tumor treatment by stimulating droplets vaporization	[115]

Light	Three-layered polyplex micelles	pDNA and photoresponsive materials	Systemic gene transfer in tumor	[116]
	Nanorods	DNA doxorubicin	Multidrugresistant cancer cells treatment	[117]
	Nanogel	Doxorubicin graphene	Diagnosis and treatment of lung cancer	[118]
	Carbon-based nanotubes	Doxorubicin	Light- and temperature-based therapy and chemotherapy	[119]
	Plasma membrane-based nanocarriers	Doxorubicin and indocyanine green	Light- and temperature-based therapy and chemotherapy	[120]

**Table 2:** Examples of different endogenous stimuli-based drug delivery options and their different carrier systems along with their application.

Stimulus	Carrier	Associated cargo	Application	Ref
Hypoxia	Mesoporous silica nanoparticles	Oligonucleotide (CpG) and chlorin e6	Cancer therapy to boost the immune system	[156]
	Upconversion nanoparticles	Tirapazamine and indocyanine green	Treatment of tumor by implementing photodynamic therapy (PDT) and chemotherapy	[157]
	Polymeric micelles	Doxorubicin	Radiotherapy and chemotherapy for treating cancer	[158]
	Albumin nanoparticles	Oxaliplatin prodrug and Ce6	PDT and chemotherapy in cancer	[159]

Redox	Polymersomes	Doxorubicin	Chemotherapy for lung carcinoma	[160]
	Nanoparticles	Paclitaxel	Drug release by triggering GSH and targeted therapy to activate tumor	[161]
	Polyphosphazene nanoparticles	Doxorubicin	Redox-stimulated chemotherapy and photo-thermal therapy	[162]
	Nanoparticles	Catalase and photosensitizer of methylene blue	Hypoxic cancer cells are treated using H2O2-responsive drug release and a light triggering mechanism	[163]

pH	Polymeric micelles	Epirubicin	Intracellular release of drug	[164]
	CaP nanocarriers	Mn^2+^	Detection of ultrasmall liver metastasis and imaging of solid tumors in addition to neutron capture therapy	[165]

**Table 3:** Example of some recent progression in receptor-mediated drug targeting along with types of cancer treated.

Type of approach	Type of cancer cell	Drug + Ligand	Feature	Ref
Transferrin receptor	A2780 ovarian carcinoma cells	DOX + *R*8 and transferrin	Flow cytometry exposed a 2-fold increase in intracellular DOX delivery in an ovarian xenograft model	[200]
	Glioma cancer lines (LN229 and U87)	Pc 4 + transferrin peptide	Cellular uptake was higher than nontargeted conjugate. Transferrin-directed gold nanoparticles were promisingly efficient for delivery of Pc4 that is used in noninvasive imaging of brain tumor	[201]
	OVCAR-3, MDA-MB231, and MDA-MB231 (R) cell lines	Dox + transferrin	Both in vitro cell lines had shrunk cellular migration and reformed the cell cycle, while the tumor-bearing mice showed amplified delivery of doxorubicin precisely to the tumor microenvironment	[202]
	Lewis lung carcinoma (LLC) cells	Dihydroartemisinin (DHA) + transferrin (TF)	Extensive cytotoxicity as well as a decline in tumor development in tumor-bearing mice compared to nonligand targeted delivery	[203]
	Lung cancer cells (A549)	Cisplatin + cytochrome c	Confocal microscopy showed retention of cytochrome c via overexpressed transferrin. In vitro study indicated cell death through the triggering of the caspase-3 enzyme. This neo conjugate did not harm healthy lung cells, which sustained an IC50 value of 50 *μ*M observed in cisplatin + cytochrome c conjugate	[204]

CDD4 receptor	Head and neck cancers, breast cancers, and liver cancers	Paclitaxel + hyaluronic acid	Enhanced cellular retention in breast cancer in comparison with macromolecule and liver cell high tumor growth reduction compared with nanoparticle	[205]

Folate receptor	KB cells	Dox + folic acid	Elevated cargo accumulation	[206]
	Ovarian cancer	Dox + folate	Cell line analysis exhibited 10.33-fold decreased IC50 in A2780 and in OVCAR3 cell 3.93 times lower than untargeted nanoparticles	[207]
	Hela cells	Dox + folate	Higher cellular uptake	[208]

EGFR receptor	SK-OV-3 tumor xenografts	siRNA and doxorubicin + EGFR antibody conjugated immunonanoparticles	Enhanced siRNA released to EGFR expressing cell after significant cell binding	[209]

Interleukin-6 receptor	Human glioma U87 cells	pDNA + I6P7 peptide	Demonstrated unique gene expression in glioma cells U-87 of humans with deeper penetration. IV administration resulted in enhanced survival time in mice with orthotopic glioma U-87	[210]

Integrins av*β*3 and *β*5 receptor	Glioblastoma multiforme (GBM)	Cilengitide + poloxamer 188-attached heparin copolymer	Terminal deoxynucleotidyl transferase biotin-dUTP Nick end labelling assay of tumor samples and Western blot and electron microscopy showed prominent apoptosis	[95]

Human epidermal growth factor receptor 2 (HER2)	HER2 positive and multidrug-resistant breast cancer cell line (BT474/MDR)	Bevacizumab and doxorubicin + antibody	Elevated concentration of doxorubicin to the nucleus as well as steady reduction in tumor size throughout 60 days	[211]

TNF receptor	HCT 116, DOX resistant MCF-7, and CAPAN-1	Dox + TNF-relatedapoptosis-inducing ligand (Apo2L/TRAIL)	Inhibition of proliferation of multiple tumor cells through cytotoxicity and apoptosis	[212]

Lectin		(DOX, chloroquine phosphate, lamivudine, triphosphate, and efavirenz) + sugar moieties	Exhibited promising outcomes in targeted drug delivery	[213–215]
